# Supplementary material for: Distal Gastrectomy for Symptomatic Stage IV Gastric Cancer Contributes to Prognosis with Acceptable Safety Compared to Gastrojejunostomy
Source: Cancers (Basel). 2022 Jan 13;14(2):388. doi: 10.3390/cancers14020388 (PMC8773932; doi:10.3390/cancers14020388)
Supplement: Supplementary file 1 [file cancers-14-00388-s001.zip › cancers-1552521-supplementary.pdf]

cancers

MDPI

Supplementary Materials

Distal Gastrectomy for Symptomatic Stage IV Gastric Cancer Contributes to Prognosis with Acceptable Safety Compared to Gastrojejunostomy

Nobuaki Fujikuni, Kazuaki Tanabe, Minoru Hattori, Yuji Yamamoto, Hirofumi Tazawa, Kazuhiro Toyota, Noriaki Tokumoto, Ryuichi Hotta, Senichiro Yanagawa, Yoshihiro Saeki, Yoichi Sugiyama, Masahiro Ikeda, Masayuki Shishida, Toshikatsu Fukuda, Keisuke Okano, Masahiro Nishihara, Hideki Ohdan
and on behalf of Hiroshima Surgical study group of Clinical Oncology (HiSCO)

a

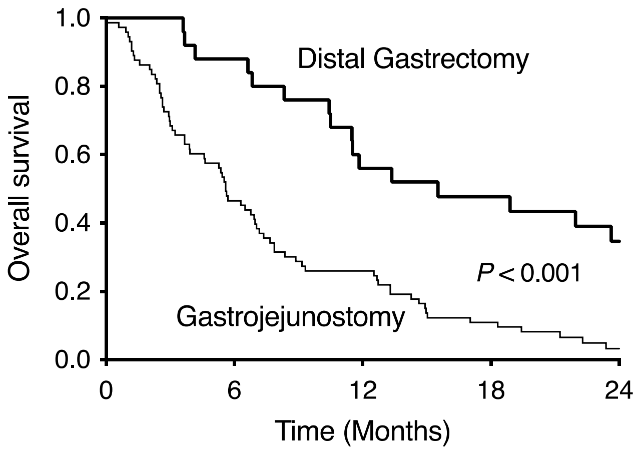

Number of patients at risk

Gastrojejunostomy	73	34	19	8	2
Distal Gastrectomy	25	22	14	11	8

b

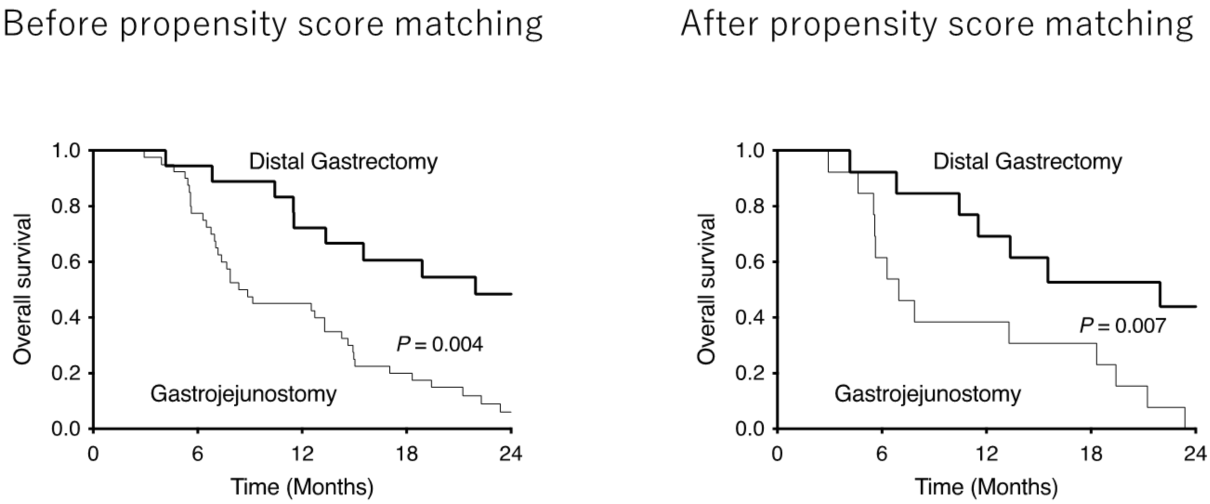

Before propensity score matching — Number of patients at risk

Gastrojejunostomy	40	31	18	8	2
Distal Gastrectomy	18	17	13	11	8

After propensity score matching — Number of patients at risk

Gastrojejunostomy	13	8	5	4	0
Distal Gastrectomy	13	12	9	6	5

c

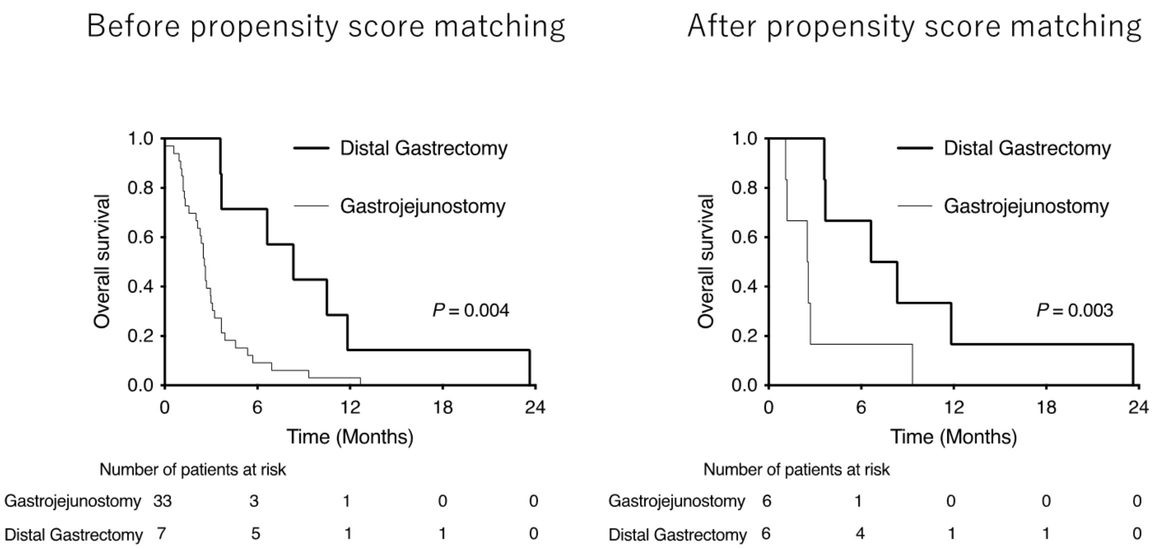

Figure S1. Kaplan–Meier survival curves of distal gastrectomy and gastrojejunostomy for stage IV gastric cancer with gastric outlet obstruction (a) all cases, (b) with chemotherapy and (c) without chemotherapy.

Table S1. General characteristics of 98 GC patients with gastric outlet obstruction.

**Variables**		**Distal gastrectomy**	**Gastrojejunostomy**	***p*-value**
		**(*n* = 25)**	**(*n* = 73)**	
Age	< 70	10 (40.0%)	26 (35.6%)	0.811
	≥ 70	15 (50.0%)	47 (64.4%)	
Sex	Male	16 (64.0%)	49 (67.1%)	0.809
	Female	9 (36.0%)	24 (32.9%)	
BMI	< 25	21 (84.0%)	65 (89.0%)	0.725
	≥ 25	4 (16.0%)	8 (11.0%)	
ASA PS	1	0 (0%)	2 (2.7%)	0.309
	2	20 (80.0%)	48 (65.8%)	
	3	5 (20.0%)	23 (31.5%)	
PS	0	12 (48.0%)	42 (57.5%)	0.336
	1	10 (40.0%)	16 (21.9%)	
	2	2 (8.0%)	10 (13.7%)	
	3	1 (4.0%)	5 (6.8%)	
Anemia	Present	12 (48.0%)	44 (60.3%)	0.351
	Absent	13 (52.0%)	29 (39.7%)	
Obstruction	Present	25 (100.0%)	73 (100.0%)	
CEA	< 5	18 (72.0%)	38 (52.8%)	0.106
	≥ 5	7 (28.0%)	34 (47.2%)	
	unknown		1	
CA19-9	< 37	14 (58.3%)	43 (59.7%)	1.000
	≥ 37	10 (41.7%)	29 (40.3%)	
	unknown	1	1	
NLR	< 3	17 (68.0%)	21 (28.8%)	**<0.001**

	≥ 3	8 (32.0%)	52 (71.2%)	
PNI	< 40	10 (40.0%)	43 (58.9%)	0.111
	≥ 40	15 (60.0%)	30 (41.1%)	
mGPS	0	7 (33.3%)	14 (20.0%)	0.241
	1–2	14 (66.7%)	56 (80.0%)	
	unknown	4	3	
Macroscopic Type	Non-4	21 (84.0%)	60 (82.2%)	1.000
	4	4 (16.0%)	13 (17.8%)	
Histologic type	Intestinal	8 (32.0%)	26 (35.6%)	0.812
	Diffuse	17 (68.0%)	47 (64.4%)	
Invasion of adjacent organs	Present	1 (4.0%)	8 (11.0%)	0.441
	Absent	24 (96.0%)	65 (89.0%)	
Distant lymph node metastasis	Present	3 (12.0%)	25 (34.2%)	**0.041**
	Absent	22 (88.0%)	48 (65.8%)	
Liver metastasis	Present	7 (28.0%)	19 (26.0%)	1.000
	Absent	18 (72.0%)	54 (74.0%)	
Peritoneal metastasis	Present	17 (68.0%)	54 (74.0%)	0.608
	Absent	8 (32.0%)	19 (26.0%)	
Number of metastasis factors	1	20 (80.0%)	44 (60.3%)	0.312
	2	4 (16.0%)	22 (30.1%)	
	3	1 (4.0%)	4 (5.5%)	
	4	0	3 (4.1%)	
Postoperative Chemotherapy	Present	18 (72.0%)	40 (54.8%)	0.161
	Absent	7 (28.0%)	33 (45.2%)	
Surgical approach	Open	21 (84.0%)	56 (76.7%)	0.577
	Laparoscopic	4 (16.0%)	17 (23.3%)	
Operative time	(min)	231 (118–366)	125 (61–268)	**<0.001**
Blood loss	(ml)	85 (10–680)	10 (0–555)	**<0.001**
Hospital stays	(days)	13 (9–138)	17 (1–72)	0.974
Complications ≥CD3	Present	2 (8.0%)	7 (9.6%)	1.000
	Absent	23 (92.0%)	66 (90.4%)	

Variables in bold are statistically significant ($p < 0.05$). BMI, body mass index; ASA PS, American Society of Anesthesiologists physical status; PS, Performance Status; CEA, carcinoembryonic antigen; CA19-9, carbohydrate antigen 19-9; NLR, neutrophil-to-lymphocyte ratio; PNI, prognostic nutritional index; mGPS, modified Glasgow Prognostic Score; CD, Clavien-Dindo Classification.

Table S2. Univariate and multivariate analysis of overall survival of 98 GC patients with gastric outlet obstruction.

Variables		Univariate HR (95%CI)	*p*-value	Multivariate HR (95%CI)	*p*-value
Age	< 70	1			
	≥ 70	1.518 (0.992–2.324)	0.054		
Sex	Male	1			
	Female	1.510 (0.974–2.342)	0.066		
BMI	< 25	1			
	≥ 25	0.737 (0.391–1.388)	0.344		
ASA PS	1–2	1			
	3	1.901 (1.207–2.995)	**0.006**	1.420 (0.872–2.312)	0.159
PS	0	1			
	1–3	1.631 (1.081–2.462)	**0.020**	0.724 (0.406–1.292)	0.274
Anemia	Present	1.112 (0.735–1.681)	0.615		
	Absent	1			
CEA	< 5	1			
	≥ 5	1.212 (0.800–1.834)	0.364		
CA19-9	< 37	1			
	≥ 37	1.214 (0.792–1.862)	0.373		
NLR	< 3	1			
	≥ 3	1.959 (1.267–3.028)	**0.002**	1.397 (0.817–2.387)	0.222
PNI	< 40	1			
	≥ 40	0.583 (0.382–0.890)	**0.012**	0.710 (0.437–1.153)	0.166
mGPS	0	1			
	1–2	1.581 (0.941–2.657)	0.084		
Macroscopic type	Non-4	1			
	4	0.918 (0.541–1.556)	0.750		
Histologic type	Intestinal	1			
	Diffuse	1.092 (0.710–1.680)	0.687		
Invasion of adjacent organs	Present	0.901 (0.448–1.813)			
	Absent	1	0.770		
Distant lymph node metastasis	Present	1.259 (0.801–1.980)	0.317		
	Absent	1			
Liver metastasis	Present	0.916 (0.573–1.464)	0.713		
	Absent	1			
Peritoneal metastasis	Present	1.564 (0.979–2.498)	0.061		
	Absent	1			
Number of metastasis factors	1	1			
	2–4	1.461 (0.955–2.236)	0.081		
Postoperative Chemotherapy	Present	0.201 (0.127–0.317)	**<0.001**	0.168 (0.088–0.319)	**<0.001**
	Absent	1			
Surgical approach	Open	1			
	Laparoscopic	1.718 (1.046–2.823)	**0.033**	1.148 (0.666–1.980)	0.619
Surgical	Distal Gastrectomy	0.383 (0.233–0.629)	**<0.001**	0.371 (0.211–0.651)	**<0.001**

procedure	Gastrojejunostomy	1			
Operative time	< 134	1			
	≥ 134	0.857 (0.571–1.286)	0.455		
Blood loss	< 20	1			
	≥ 20	0.880 (0.584–1.325)	0.540		
Hospital stays	< 16	1			
	≥ 16	1.883 (1.237–2.865)	**0.003**	1.722 (1.055–2.810))	**0.030**
Complications ≥CD3	Present	1.574 (0.787–3.146)	0.200		
	Absent	1			

HR, hazard ratio; CI, confidential index. Variables in bold are statistically significant ($p < 0.05$).

Table S3. General characteristics before propensity score matching of 98 GC patients with gastric outlet obstruction.

		**With chemotherapy**			Without chemotherapy		
**Variables**		**Distal gastrectomy**	**Gastrojejunostomy**	*p*-value	Distal gastrectomy	Gastrojejunostomy	*p*-value
		**(*n* = 18)**	**(*n* = 40)**		(*n* = 7)	(*n* = 33)	
Age	< 70	9 (50.0%)	17 (42.5%)	0.402	1 (14.3%)	9 (27.3%)	0.656
	≥ 70	9 (50.0%)	23 (57.5%)		6 (85.7%)	24 (72.7%)	
Sex	Male	14 (74.8%)	29 (72.5%)	0.756	2 (28.6%)	20 (60.6%)	0.211
	Female	4 (22.2%)	11 (27.5%)		5 (71.4%)	13 (39.4%)	
BMI	< 25	15 (83.3.3%)	35 (87.5%)	0.694	6 (85.7%)	30 (90.9%)	1.000
	≥ 25	3 (16.7%)	5 (12.5%)		1 (14.3%)	3 (9.1%)	
ASA PS	1	0 (0%)	1 (2.5%)	0.519	0 (0%)	1 (3.0%)	1.000
	2	16 (88.9%)	29 (72.5%)		4 (57.1%)	19 (57.6%)	
	3	2 (11.1%)	10 (25.0%)		3 (42.9%)	13 (39.4%)	
PS	0	10 (55.6%)	33 (82.2%)	0.072	2 (28.6%)	9 (27.3%)	0.942
	1	7 (38.9%)	5 (12.5%)		3 (42.9%)	11 (33.3%)	
	2	1 (5.6%)	2 (5.0%)		1 (14.3%)	8 (24.2%)	
	3	0 (0%)	0 (0%)		1 (14.3%)	5 (15.2%)	
Anemia	Present	9 (50.0%)	20 (50.0%)	1.000	3 (42.9%)	24 (72.7%)	0.187
	Absent	9 (50.0%)	20 (50.0%)		4 (57.1%)	9 (27.3%)	
Obstruction	Present	18 (100.0%)	40 (100.0%)	-	7 (100.0%)	33 (100%)	-
	Absent	0	0		0	0	
CEA	< 5	14 (77.8%)	22 (56.4%)	0.149	4 (57.1%)	16 (48.5%)	1.000
	≥ 5	4 (22.2%)	17 (43.6%)		3 (42.9%)	17 (51.5%)	
	unknown	0	1		0	0	
CA19-9	< 37	11 (64.7%)	23 (59.0%)	0.772	3 (42.9%)	20 (66.6%)	0.432
	≥ 37	6 (35.3%)	16 (41.0%)		4 (57.1%)	13 (39.4%)	
	unknown	1	1		0	0	
NLR	< 3	11 (61.1%)	16 (40.0%)	0.163	6 (85.7%)	5 (15.2%)	**<0.001**
	≥ 3	7 (38.9%)	24 (60.0%)		1 (14.3%)	28 (84.8%)	
PNI	< 40	6 (33.3%)	22 (55.0%)	0.161	4 (57.1%)	21 (63.6%)	1.000
	≥ 40	12 (66.7%)	18 (45.0%)		3 (42.9%)	12 (36.4%)	
mGPS	0	5 (35.7%)	7 (18.9%)	0.272	2 (28.6%)	7 (21.2%)	1.000
	1–2	9 (64.3%)	30 (81.1%)		5 (71.4%)	26 (78.8%)	

	unknown	4	3		0	0	
Macroscopic type	Non-4	16 (88.9%)	32 (80.0%)	0.483	5 (71.4%)	28 (84.8%)	0.584
	4	2 (11.1%)	8 (20.0%)		2 (28.6%)	5 (15.2%)	
Histologic type	Intestinal	6 (33.3%)	13 (32.5%)	1.000	2 (28.6%)	13 (39.4%)	0.691
	Diffuse	12 (66.7%)	27 (67.5%)		5 (71.4%)	20 (60.6%)	
Invasion of adjacent organs	Present	1 (5.6%)	4 (10.0%)	0.670	0 (0%)	4 (12.1%)	0.582
	Absent	17 (94.4%)	36 (90.0%)		7 (100%)	29 (87.9%)	
Distant lymph node metastasis	Present	2 (11.1%)	17 (42.5%)	**0.032**	1 (14.3%)	8 (24.2%)	0.672
	Absent	16 (88.9%)	23 (57.5%)		6 (85.7%)	25 (75.8%)	
Liver metastasis	Present	5 (27.8%)	11 (27.5%)	1.000	2 (28.6%)	8 (24.2%)	1.000
	Absent	13 (72.2%)	29 (72.5%)		5 (71.4%)	25 (75.8%)	
Peritoneal metastasis	Present	12 (66.7%)	26 (65.0%)	1.000	5 (71.4%)	28 (84.8%)	0.584
	Absent	6 (33.3%)	14 (35.0%)		2 (28.6%)	5 (15.2%)	
Number of metastasis factors	1	15 (83.3%)	24 (60.0%)	0.319	5 (71.4%)	20 (60.6%)	0.478
	2	3 (16.7%)	12 (30.0%)		1 (14.3%)	10 (30.3%)	
	3	0 (0%)	3 (7.5%)		1 (14.3%)	1 (3.0%)	
	4	0 (0%)	1 (2.5%)		0	2 (6.1%)	
Surgical approach	Open	18 (100%)	32 (80.0%)	0.093	3 (42.9%)	24 (72.7%)	0.187
	Laparoscopic	0 (0%)	8 (20.0%)		4 (57.1%)	9 (27.3%)	

Variables in bold are statistically significant ($p < 0.05$).

Table S4. General characteristics after propensity score matching of 98 GC patients with gastric outlet obstruction.

		With chemotherapy			Without chemotherapy		
**Variables**		**Distal gastrectomy**	**Gastrojejunostomy**	***p*-value**	**Distal gastrectomy**	**Gastrojejunostomy**	***p*-value**
		**(*n* = 13)**	**(*n* = 13)**		**(*n* = 6)**	**(*n* = 6)**	
Age	< 70	5 (38.5%)	5 (38.5%)	1.000	0 (0%)	1 (16.7%)	1.000
	≥ 70	8 (61.5%)	8 (61.5%)		6 (100%)	5 (83.3%)	
Sex	Male	10 (76.9%)	9 (69.2%)	1.000	1 (16.7%)	4 (66.7%)	0.242
	Female	3 (23.1%)	4 (30.8%)		5 (83.3%)	2 (33.3%)	
BMI	< 25	11 (84.6%)	12 (92.3%)	1.000	5 (83.3%)	5 (83.3%)	1.000
	≥ 25	2 (15.4%)	1 (7.2%)		1 (16.7%)	1 (16.7%)	
ASA PS	1	0 (0%)	0 (0%)	1.000	0 (0%)	1 (16.7%)	0.393
	2	11 (84.6%)	12 (92.3%)		4 (66.7%)	2 (33.3%)	
	3	2 (15.4%)	1 (7.7%)		2 (33.3%)	3 (50.0%)	
PS	0	7 (53.8%)	11 (84.6%)	0.169	1 (16.7%)	0	0.757
	1	5 (38.5%)	1 (7.7%)		3 (50.0%)	4 (66.7%)	
	2	1 (7.7%)	1 (7.7%)		1 (16.7%)	1 (16.7%)	
	3	0 (0%)	0 (0%)		2 (33.3%)	1 (16.7%)	
Anemia	Present	8 (61.5%)	8 (61.5%)	1.000	2 (33.3%)	4 (66.7%)	0.567
	Absent	5 (38.5%)	5 (38.5%)		4 (66.7%)	2 (33.3%)	
Obstruction	Present	13 (100.0%)	13 (100.0%)	-	6 (100.0%)	6 (100.0%)	-

	Absent	0	0		0	0	
CEA	< 5	10 (76.9%)	5 (38.5%)	0.111	4 (66.7%)	2 (33.3%)	0.567
	≥ 5	3 (23.1%)	8 (61.5%)		2 (33.3%)	4 (66.7%)	
CA19-9	< 37	10 (76.9%)	7 (53.8%)	0.411	3(50.0%)	3 (50.0%)	1.000
	≥ 37	3 (23.1%)	6 (46.2%)		3(50.0%)	3 (50.0%)	
NLR	< 3	9 (69.2%)	5 (38.5%)	0.238	5 (83.3%)	5 (83.3%)	1.000
	≥ 3	4 (30.8%)	8 (61.5%)		1 (16.7%)	1 (16.7%)	
PNI	< 40	5 (38.5%)	8 (61.5%)	0.434	3(50.0%)	2 (33.3%)	1.000
	≥ 40	8 (61.5%)	5 (38.5%)		3(50.0%)	4 (66.7%)	
mGPS	0	5 (38.5%)	2 (15.4%)	0.378	2(33.3%)	1 (16.7%)	1.000
	1–2	8 (61.5%)	11 (84.6%)		4 (66.7%)	5 (83.3%)	
Macroscopic type	Non-4	12 (92.3%)	10 (76.9%)	0.593	4 (66.7%)	5 (83.3%)	1.000
	4	1 (7.7%)	3 (23.1%)		2(33.3%)	1 (16.7%)	
Histologic type	Intestinal	4 (30.8%)	4 (30.8%)	1.000	1 (16.7%)	0	1.000
	Diffuse	9 (69.2%)	9 (69.2%)		5 (83.3%)	6 (100.0%)	
Invasion of adjacent organs	Present	1 (7.7%)	2 (15.4)	1.000	0	2 (33.3%)	0.455
	Absent	12 (92.3%)	11 (84.6%)		6 (100.0%)	4 (66.7%)	
Distant lymph node metastasis	Present	2 (15.4%)	2 (15.4%)	1.000	1 (16.7%)	2 (33.3%)	1.000
	Absent	11 (84.6%)	11 (84.6%)		5 (83.3%)	4 (66.7%)	
Liver metastasis	Present	3 (23.1%)	5 (38.5%)	0.673	1 (16.7%)	1 (16.7%)	1.000
	Absent	10 (76.9%)	8 (61.5%)		5 (83.3%)	5 (83.3%)	
Peritoneal metastasis	Present	8 (61.5%)	9 (69.2%)	1.000	5 (83.3%)	4 (66.7%)	1.000
	Absent	5 (38.5%)	4 (30.8%)		1 (16.7%)	2 (33.3%)	
Number of metastasis factors	1	11 (84.6%)	9 (69.2%)	0.497	4 (66.7%)	3 (50.0%)	0.480
	2	2 (15.4%)	3 (23.1%)		1 (16.7%)	2 (33.3%)	
	3	0 (0%)	0 (0%)		1 (16.7%)	0	
	4	0 (0%)	1 (7.7%)		0	1 (16.7%)	1.000
Surgical approach	Open	13 (100%)	11 (84.6%)	0.480	3 (50.0%)	4 (66.7%)	
	Laparoscopic	0	2 (15.4%)		3 (50.0%)	2 (33.3%)	
